# Evidence-Based Public Health 2017

**DOI:** 10.1155/2017/2607397

**Published:** 2017-11-15

**Authors:** Giedrius Vanagas, Malgorzata Bala, Stefan K. Lhachimi

**Affiliations:** ^1^Department of Preventive Medicine, Lithuanian University of Health Sciences, LT-49264 Kaunas, Lithuania; ^2^Department of Hygiene and Dietetics, Jagiellonian University Medical College, 31-034 Krakow, Poland; ^3^Cooperative Research Group for Evidence-Based Public Health (EBPH), Institute for Public Health and Nursing Research, University of Bremen and Leibniz Institute for Prevention Research and Epidemiology-BIPS, Achterstraße 30, 28359 Bremen, Germany; ^4^Health Sciences Bremen, University of Bremen, Bibliothekstraße 1, 28359 Bremen, Germany

Public health science has made considerable advances in its aim to improve scientific standards in order to generate well-grounded evidence. One of the principles to promote higher standards in public health is methodological development in the field of evidence-based public health (EBPH) [1–6]. EBPH can be defined as a process of integrating evidence from scientific research and practice to improve the health of the target population.

The key components of EBPH include making decisions on the basis of the best available scientific evidence and using sound data collection and research methods together with engaging the community in the decision-making. An EBPH approach could potentially have numerous direct and indirect benefits, including access to more and higher-quality information on best practice, a higher likelihood of successful prevention programs and policies, greater workforce productivity, and more efficient use of public and private resources.

The most critical problem for EBPH is the absence of evidence and the lack of a conceptual framework regarding how much evidence is sufficient to judge and evaluate our policy decisions. Often, we have evidence that something should be done (e.g., needs of assessment, measures of prevalence, and preventability of risks and conditions) but, in most cases, we lack evidence regarding what should be done (e.g., the effectiveness of health intervention) or how to do it (e.g., evaluation of the health process) [7]. From this viewpoint, our special issue has addressed some important aspects and shows the increasing importance of EBPH as a research field in its own right.

The study by A. Berke-Berga et al. provided evidence for the development of insightful health policies in the case of Latvia. Their study examined the evolution of distributional differences in perceived health status in recent years and, based on the empirical evidence, the authors concluded that a favorable health inequality index does not confirm a reduced burden of unavoidable inequalities in health in the worse-off group of the population and the relative contributions of SES-related determinants to the production of change in health inequalities over time. Their study generates evidence for insightful health policy development.

The economic impact of cancer is enormous for both the person with cancer and for society as a whole. Vietnam is now implementing the National Strategy for Cancer Control up to 2020. Cancer prevention and control in Vietnam are still facing several challenges, such as a lack of comprehensive actions from the involved stakeholders, the unavailability of services for cancer screening, and early detection at the grassroots level of care, as well as a shortage of human capacities and financial resources. Households belonging to the poor or poorest socioeconomic groups are significantly associated with increased impoverishment due to their healthcare costs related to treatment [8]. The study by V. M. Hoang et al. generates new evidence regarding the financial burden on households and the impact of poverty on cancer treatment in Vietnam. Given the evidence, policy actions that can reduce/remove the financial barriers and provide financial protection for cancer patients (as well as other population groups) are urgently required.

The study by K.-S. Bang et al. explored the health status and health-related quality of life of rural elderly Vietnamese and assessed their needs for healthcare services. The results of this study reveal epidemiological patterns in Vietnam, shifting from a predominance of communicable diseases to noncommunicable diseases. The role of the healthcare provider in rural areas should be strengthened to effectively address the need for healthcare services of the elderly population, as well as appropriate health education to promote healthy lifestyles. Further research is required to promote evidence-based health policy development for chronic illness management programs for the rural elderly in Vietnam.

Developing countries face the dual burden of both undernutrition and overnutrition simultaneously, exerting substantial strain on the already overburdened health systems. The prevalence of overweight and obesity has increased manyfold in Asia, especially in South Asia. The study by I. Khan et al. found a significant dose-response relationship of increasing comorbidities with increasing weight. The association has important implications for public health planning and management, as the health effects of obesity at the individual and community levels manifest through these comorbid states, which are often known to increase in proportion with increasing weight.

Climbers, workers, and tourists who travel to high-altitude destinations are at risk of altitude illness due to hypoxia. The most common form of altitude illness is acute mountain sickness (AMS). Previous epidemiological studies on the relationship between smoking and AMS risk yielded inconsistent findings. Therefore, a meta-analysis of observational studies (cross-sectional studies, case-control, or cohort studies) was performed by C. Masuet-Aumatell et al. to determine whether smoking was related to the development of AMS. The meta-analysis contains 11 full-text studies on smoking and AMS, including 7 cross-sectional studies, 3 cohort studies, and 1 case-control study.

This annual issue continues our efforts to provide relevant evidence and tools for public healthcare practitioners. Finally, with this issue we are aiming to stimulate the application of evidence-based knowledge to the practice of public health and public health decision-making.
